# Parasitic dermoid cyst managed laparoscopically in a 29-year-old woman: a case report

**DOI:** 10.1186/1752-1947-3-63

**Published:** 2009-02-16

**Authors:** Ciprian E Bartlett, Ashfaq Khan, Narendra Pisal

**Affiliations:** 1 Obstetrics and Gynaecology, Homerton University Hospital NHS Foundation Trust, Homerton Row, London, UK; 2 Obstetrics and Gynaecology, Whittington Hospital, Magdala Avenue, London, UK

## Abstract

**Introduction:**

Dermoid cysts are one of the most frequently occurring ovarian cysts; parasitic dermoid cysts, however, are extremely rare.

**Case presentation:**

We report a case of a 29-year-old Japanese woman with an incidental finding of an adnexal mass on bimanual examination. Sonographic imaging reported an 8 cm mass in the Pouch of Douglas. This was found to be a parasitic dermoid cyst and was removed at laparoscopy.

**Conclusion:**

We believe laparoscopy to be a safe and effective means of managing parasitic ovarian dermoid cysts.

## Introduction

Dermoid cyst (benign cystic teratoma) of the ovary is one of the most common ovarian neoplasms but parasitic cysts are very rare and their true incidence is unknown. We report a case of a parasitic dermoid cyst in the Pouch of Douglas (POD) managed laparoscopically. To the best of our knowledge, this is the only reported case of laparoscopic removal of a non-omental parasitic ovarian dermoid in the literature.

## Case presentation

A 29-year-old Japanese woman underwent a bimanual pelvic examination, under anaesthesia, prior to large-loop excision of the transformation zone for large volume high-grade cervical intraepithelial neoplasia. During this examination, a 10 cm right adnexal mass was noted. Outpatient pelvic ultrasonography (USS) and cancer antigen 125 (CA-125) were arranged.

On review, the patient was asymptomatic and the USS showed a normal uterus and left ovary. The right ovary was not positively identified. There was a 6 × 8 cm echogenic mass seen on the left and posterior to the fundus of the uterus with no abnormal vascular flow. It was uncertain whether this mass was fibroid, ovarian or bowel in origin. The CA-125 was mildly elevated at 38 (normal range 0–35 u/ml). The plan was to review her with magnetic resonance imaging (MRI) of the pelvis and tumour markers; CA-125, CA-19-9, carcino-embyonic antigen (CEA), alpha-feto protein, and human chorionic gonadotropin. The patient failed to attend for the MRI. The tumour markers were all normal except for a slightly elevated CEA of 7 ug/L (normal range 0–4 ug/mL). She was then booked for an elective laparoscopy.

Laparoscopy showed a normal uterus, left ovary and tube, a dilated right tube and a small right ovary. A 6 × 6 cm mass was noted in the POD adherent to the posterior aspect of the uterus on the left side. No ligamentous attachment or vascular pedicle was seen. Adhesiolysis was performed by blunt dissection, and the cyst was decompressed within a bag and removed through a 10 mm suprapubic port. The patient's recovery was uneventful and she was discharged after 24 hours.

Histopathology confirmed a mature cystic teratoma containing squamous epithelium and mature skin adnexal structures.

## Discussion

Mature cystic teratoma is one of the most common ovarian tumours, accounting for 10% to 20% of all ovarian neoplasms. Teratomas are derived from germ cells and may contain structures from all three embryonic germ layers. They are bilateral in 8% to 15% of cases [1]. Teratomas may occur at other sites within the body, usually midline or paramedian structures. These sites may be sacral, testicular, mediastinal or retroperitoneal [2].

Parasitic dermoid cysts are extremely rare entities, and their actual incidence is unknown. Several theories exist to explain their occurrence. The first theory proposes the development within a supernumerary or ectopic ovary, which may occur after implantation of ovarian tissue after surgical procedure or inflammation such as pelvic inflammatory disease. Supernumerary ovaries may also occur as a result of abnormal arrest of germinal cells in the dorsal mesentery during their embryonic migration to the genital ridge [3]. Another theoretical mechanism is autoamputation and reimplantation of the dermoid cyst as a result of torsion. If torsion of the tumour is subacute, an inflammatory response may occur which causes the tumour to become adherent to surrounding structures and neovascularisation may occur. The tumour may then become parasitic by detaching itself from its original blood supply [4]. The right ovary of our patient appeared very small, therefore making it very likely that this might have been the origin of the dermoid cyst; that is, autoamputation and re-implantation the likely mechanism of origin. We were unable to explain the viability of this cyst in light of an absence of an obvious vascular pedicle.

Pre-operative assessment of these adnexal masses using clinical signs and symptoms, tumour markers and pelvic ultrasound is essential to establish risk of malignancy and therefore plan an appropriate surgical approach [5]. Management of these cysts may be by laparotomy or laparoscopy; however, laparoscopic cystectomy should be the procedure of choice for parasitic dermoid cysts if the clinical evaluation suggests a benign lesion [6]. Laparoscopy offers several benefits, including reduced blood loss, postoperative pain, shorter hospital stay and quicker recovery [1,6]. Caution is required when removing dermoid cysts, as spillage of sebaceous material can result in chemical granulomatous peritonitis, which has an incidence of approximately 0.2%. This risk can be reduced by decompression of the cyst within an impermeable bag prior to removal.

## Conclusion

Laparoscopy is a safe and effective method of managing parasitic dermoid cysts, provided preoperative assessments are suggestive of a benign tumour.

## Abbreviations

POD: Pouch of Douglas; USS: ultrasonography; CA-125: cancer antigen 125; MRI: magnetic resonance imaging; CEA: carcino-embyonic antigen

## Consent

Written informed consent was obtained from the patient for publication of this case report. A copy of the written consent is available for review by the Editor-in-Chief of this journal.

## Competing interests

The authors declare that they have no competing interests.

## Authors' contributions

CEB conducted the literature review and conceived and drafted the manuscript. AK helped in collecting records and preparing the manuscript. NP critically revised the manuscript for intellectual content and gave final approval of the version for publication. All authors revised and approved the manuscript.
